# Internalizing Pathways to Adolescent Substance Use from Adverse Childhood Experiences

**DOI:** 10.3390/ijerph21111408

**Published:** 2024-10-24

**Authors:** Michelle G. Villar, Nicole M. Fava, Robert A. Zucker, Elisa M. Trucco

**Affiliations:** 1 Wien Center for Alzheimer’s, Mount Sinai Medical Center, 4300 Alton Rd, Miami, FL 33140, USA; 2 Center for Children and Families, Florida International University, 11200 SW 8th Street, Miami, FL 33139, USA; etrucco@fiu.edu; 3 Robert Stempel College of Public Health & Social Work, Florida International University, 11200 SW 8th Street, Miami, FL 33139, USA; 4 Department of Psychiatry, University of Michigan, 4250 Plymouth Road, Ann Arbor, MI 48109, USA; zuckerra@med.umich.edu; 5 Department of Psychology, Florida International University, 11200 SW 8th Street, Miami, FL 33199, USA

**Keywords:** adverse childhood experiences, ACEs, internalizing problems, depression, anxiety, somatic, substance use, adolescence, adolescents

## Abstract

The mediating role of anxious, depressive, and somatic symptoms was examined in the association between adverse childhood experiences (ACEs) and adolescent substance use, with attention to the unique effects of each set of symptoms within the same model. Adolescents (n = 701) were assessed over time (ages 3–17) in a majority male (70.5%) and white (89.9%) sample. Findings indicate that depressive symptoms mediated the association between ACEs and adolescent cigarette and marijuana use. Although significant indirect effects remained when accounting for externalizing behavior, a novel protective pathway emerged through parent-reported youth anxiety and alcohol use. Assessing internalizing symptoms as separate facets within the same model is critical if we are to inform prevention programs that are tailored to the individual needs of youth who have experienced ACEs.

## 1. Introduction

Prior research on childhood adversity has supported an association between adverse childhood experiences (ACEs) and maladaptive outcomes across the lifespan, including during adolescence [1,2,3]. ACEs are traumatic and/or negative events that occur before someone is 18 years old (e.g., abuse and neglect, domestic violence, divorce, and incarceration [4]). ACEs typically co-occur [4,5] in a graded association with negative outcomes [4,6]. The majority of people in the US experience at least one ACE in their lifetime, with just about half of the children in the US experiencing at least one ACE and 1 in 10 children experiencing three or more ACEs [7]. More specifically, cumulative ACEs [4] have been linked in a graded association with negative outcomes in later childhood and adolescence, such as depressive and anxious symptomatology [8] and substance use (SU) [9,10], across multiple populations and contexts. Yet, while ACEs have been linked to many negative outcomes, little work has examined the developmental associations that may contribute to these connections. There is a relative dearth of studies examining the association between ACEs and adolescent SU via internalizing symptomatology (e.g., anxious, depressive, and somatic symptoms). Moreover, the operationalization of this association varies between studies, thus obfuscating the precise nature of the mediating relation. Some researchers utilize observer ratings in the form of parent reports [11], while others utilize subjective evaluations via adolescent self-reports [12]. A greater understanding of the precise mechanisms through which ACEs lead to adolescent SU can inform tailored prevention programming for youth with histories of adversity and trauma who are at increased risk of negative outcomes, including SU. Our goal in the current study was to investigate the mediated effect of ACEs on adolescent SU via internalizing symptomatology, with a specific focus on the precise nature of these internalizing behaviors, disaggregated into depressive, anxious, and somatic symptom sets.

### 1.1. Impact of ACEs on Substance Use

Substance use is a widely noted negative outcome linked to ACEs throughout the lifespan [4,10,13]. For example, researchers of the original ACEs study [4] reported a two- to ten-fold increase in SU (i.e., alcohol, cigarettes, and illicit drugs) among adults as the number of ACEs increased. More recently, similar patterns have been evidenced among adolescents and young adults, suggesting that ACEs are related to the onset of SU during adolescence [8,9]. For example, Dube et al. [13] demonstrated that nearly all individual ACEs measured in their study (e.g., emotional, physical, and sexual abuse, emotional and physical neglect, and living with people with a SU problem or mental illness) increased the risk of lifetime alcohol use among adolescents. Additionally, a person’s total ACE score had a statistically significant graded relation with initiating alcohol use during early adolescence [13]. These findings support a strong graded association between cumulative ACEs and SU during adolescence. From a developmental perspective, adolescence represents a time characterized by significant maturational changes in biological and social systems, setting the stage for the potential vulnerability to SU [14,15]. Thus, we were interested in determining a mechanism for this empirically supported association between ACEs and adolescent SU.

### 1.2. Impact of ACEs on Internalizing Symptoms

While prior work has shown that ACEs are associated with adolescent SU, more research is needed to understand the pathways through which this association develops. A potential mediator may be internalizing symptoms (e.g., depressive, anxious, and somatic symptoms), as research supports links between ACEs and internalizing problems (e.g., [4,8]) and internalizing problems and adolescent SU (e.g., [16,17,18,19,20]). Furthermore, internalizing problems have been found to increase with age from 3 to 14 years old, with ACEs reported at age 3 being significantly associated with internalizing problems at age 14 [21]. For example, adolescents who experienced ACEs exhibited a dose–response pattern with mental health outcomes, such that youth reporting no ACEs also reported fewer internalizing problems (i.e., problems with peers and emotional problems) compared to youth with three or more ACEs [21]. Similarly, adolescents receiving outpatient mental health services who had also experienced multiple ACEs reported higher levels of internalizing symptoms (i.e., anxiety, depression, withdrawal, and somatization) compared to adolescents receiving the same services without histories of ACEs [22]. Additionally, among juvenile offenders, ACEs significantly predicted internalizing symptoms, more specifically, depressive disorders, suicidality, post-traumatic stress disorder, and anxiety disorders [23]. The link between ACEs and internalizing symptoms may develop as the result of prolonged and frequent adversity in the absence of mitigating social–emotional buffers (i.e., toxic stress), elevating the natural stress response and negatively impacting development during critical developmental periods [24,25]. The confluence of toxic stress and dynamic developmental changes and transitions in adolescence (e.g., puberty and structural brain maturation) may lead to heightened internalizing symptoms and disorders [26,27,28,29]. Indeed, prior work supports a link between ACEs and neurodevelopment [30], which can disrupt emotion regulation development, which may enhance the risk for internalizing problems (i.e., depression and anxiety; [31,32,33,34,35]). When coupled with existing evidence of an internalizing etiological pathway to SU via deficits in emotional processing [17,36], there is precedence for considering internalizing pathways between ACEs and SU among adolescents.

### 1.3. Internalizing Symptoms and Substance Use

A longstanding theory in the SU field, the self-medication hypothesis, posits that people may use substances to cope with problems such as stress, anxiety, or depression [37]. Namely, SU is thought to arise after the development of internalizing symptoms or stress to alleviate these unwanted clinical symptoms. Indeed, despite (1) more evidence linking externalizing problems and SU (compared to internalizing problems) and (2) the challenges associated with measurement reliability [38], research does support an association between internalizing symptoms and SU among adolescents (e.g., [16,17,18]). For example, some researchers found evidence of a positive association between depressive symptoms (e.g., internalizing symptoms) and alcohol use among children and adolescents, even after controlling for factors such as exposure to violence and parental psychopathology [39]. Similarly, Tomlinson and Brown [40] found eighth graders with current depressive symptoms (e.g., internalizing symptoms) to engage in heavier and more frequent drinking than their peers who did not experience depressive symptoms. In a longitudinal study of over 4000 high school students, those with depressive symptoms (e.g., internalizing symptoms) reported increased cigarette, marijuana, and illicit drug usage [41]. Interestingly, the authors of a review paper observed depressive symptoms (e.g., internalizing symptoms) to be consistent predictors of adolescent SU, but other negative affect symptoms (i.e., anxiety and internalizing broadly) were not as consistent [17]. When using a different operationalization of internalizing symptoms, other researchers have found social fears, social phobia, and social anxiety to all be significantly associated with nicotine dependence, even after controlling for comorbid depression, in a sample of adolescents and young adults [42]. However, to make reliable conclusions about the role of internalizing problems in relation to adolescent SU, consistent operationalizations must be implemented. Thus, we will examine the distinct pathways for the unique categories of internalizing symptoms (i.e., depression, anxiety, and somatic symptoms) to help better understand how ACEs impact an adolescent’s risk for using substances.

### 1.4. Impact of ACEs on Substance Use via Internalizing Symptoms

Existing attempts to investigate the link between ACEs and adolescent SU via internalizing symptoms as a mediator, while important, present inconsistent findings and methods. For example, no causal relations were found when Fishbein and colleagues [43] examined depression (e.g., internalizing symptom) as a mediating factor between illicit drug use and prior childhood trauma, whereas Douglas et al. [44] did find statistically significant evidence of a causal chain linking ACEs to increased risk of mood and anxiety disorders (e.g., internalizing symptoms), which increased the risk of substance dependence. Similarly, in one study with Black youth, the researchers operationalized internalizing symptoms as psychological distress [43] and reported that ACEs predicted psychological distress, which, in turn, predicted SU and delinquency. These different domains of internalizing symptoms, or psychological distress, were not examined separately. Using the National Longitudinal Study of Adolescent to Adult Health dataset, researchers [45] found the association between ACEs and non-medical prescription opioid use to be mediated by numerous internalizing and externalizing factors (e.g., depressive and anxious symptoms, suicidality, delinquency, risk-taking, and impulsivity). However, these researchers used data measured concurrently to represent ACEs, internalizing and externalizing symptoms, and opioid use. Thus, temporal precedence could not be considered. In the current study, we use longitudinal data to assess anxious, depressive, and somatic symptoms in the same model to determine whether a specific set of symptoms is driving the association between internalizing symptoms and adolescent SU.

Prior work has also predominantly focused on internalizing symptoms as a mediating pathway between one specific type of ACE—child maltreatment (i.e., physical abuse, sexual abuse, psychological abuse, and neglect)—and SU, rather than a broad conceptualization of cumulative ACEs. Indeed, evidence suggests that internalizing symptoms may mediate the association between child maltreatment and later SU. For example, Shin and colleagues found that depression symptoms mediated the association between childhood maltreatment and problem drinking in young adulthood [46]. Another study showed childhood maltreatment prospectively predicted cigarette use at age 16 via internalizing symptoms at age 14 [47]. Whilst looking at single adversities may be informative in teasing out relevant mechanisms, the effects of that adversity might be confounded by the experience of other adversities that have not been accounted for in the analyses. A comprehensive construct such as ACEs that includes maltreatment but is not limited to those four interpersonal experiences (i.e., sexual, emotional, and physical abuse, and neglect), may account for broader and interrelated links in the development of adolescent SU. Investigating a broad measure of ACEs is important given (1) that stressors are often highly correlated and (2) there is evidence for a graded association between cumulative ACEs and later maladaptive outcomes. ACEs tend to co-occur, such that children who have been exposed to one ACE have likely experienced others [48]. Accordingly, one-fourth of the sample in the original ACEs study reported two or more ACEs, and those who experienced four or more had at least a two-fold increase in SU [4]. Examining ACEs separately may underestimate the cumulative potential for ACEs to interfere with normative development and overestimate the associations between particular ACEs and psychological disorders; therefore, the importance of examining the effects of ACEs simultaneously has been underscored in previous research [49].

Another study design factor that may contribute to equivocal findings is the type of data collected (i.e., collateral versus self-reports). While notable strengths exist for each type of data collection, the field lacks consensus on which is more reliable. Prior work indicates that internalizing symptoms are best reported by the adolescent compared to a caregiver or teacher [50]. This may partially be due to the nature of internalizing symptoms, which primarily occur within the adolescent’s mind, as opposed to externalizing symptoms, which can be more easily observed by others (i.e., rule-breaking, aggression, etc.). However, collecting collateral data can allow for validation across multiple reporters and reduce the possible bias associated with shared method variance. For example, two groups of researchers reported similar results, indicating a pathway from internalizing symptoms to SU; however, Wu and colleagues [39] combined parent and self-report data of internalizing symptoms, while Sonntag and colleagues [42] utilized only self-report data. In addition, Duprey et al. [11] examined parent-reported data on adolescents’ internalizing problems (i.e., combined withdrawn, anxious/depressed, and somatic subscales), which mediated the link between the severity of neglect in early childhood and adolescent-reported data on SU. The use of different reporters can make it difficult to compare results across studies. We align with the belief that solely relying on self-report data introduces unnecessary limitations; therefore, in the current study, we utilize both parent and child reports of internalizing symptoms to redress the limitations of prior work focusing only on self-reports.

### 1.5. Current Study

The current study expands upon previous research in multiple ways. We used data from the Michigan Longitudinal Study (MLS) to consider the different domains of internalizing symptoms (i.e., depression, anxiety, and somatic symptoms) as distinct pathways between ACEs and adolescent SU. Previous investigations utilizing this dataset have only considered an externalizing mediating pathway between childhood adversity and adolescent SU [51] and total internalizing symptoms and coping expectancy as mediators between a specific ACE (i.e., parental violence) and a specific SU outcome (i.e., adolescent alcohol use [52]). Second, we compared the findings between self-reports and parent reports of internalizing symptoms. Third, separate models across substances that are traditionally initiated during adolescence (i.e., alcohol, cigarettes, and marijuana) were tested to determine whether the risk pathways were relevant to specific substances.

Due to previous support for significant associations between depression [49] and anxiety [42] with ACEs and adolescent SU, we hypothesized that participants reporting more ACEs will also report more anxious and depressive symptoms, and, in turn, they will also report higher levels of SU. Given the mixed results in prior work examining the impact of depression and anxiety on SU, hypotheses were not made regarding which set of symptoms would have a greater impact on adolescent SU. There is a lack of evidence regarding the impact of somatic symptoms; therefore, we did not make a specific hypothesis regarding somatic symptoms as a mediator between ACEs and adolescent SU. By separating out these facets of internalizing symptoms and testing their effects in the same model, a greater understanding of which distinct aspects of internalizing symptomatology predict adolescent SU may emerge. We also hypothesized that both self- and parent-report models will show evidence of mediation, but that self-reported symptoms will have stronger effects on the association between ACEs and adolescent SU, given the internal and personal experience of depressive and anxious symptoms [50]. Finally, since ACEs have been linked to the use of all three substances in adolescence, we did not make hypotheses about which substances may have the strongest link in this mediated pathway.

## 2. Method

### 2.1. Participants

Participants included 701 adolescents enrolled in the Michigan Longitudinal Study (MLS; [51]). The MLS is the longest running (34 years) and developmentally earliest (beginning at age 3) longitudinal family study on SU initiation in the extant literature. It followed a community-recruited sample of high-risk and community contrast families. High-risk families were identified via the courts in a 4-county-wide area. Families were recruited via the biological father, who was required, at minimum, to have a 3–5-year-old male child (identified as the male target child [MTC]), currently coupled with the son’s biological mother, and convicted of drunk driving with a high enough blood alcohol level to be de facto evidence of their satisfying the criteria for alcohol use disorder (AUD). These 3 family members were enrolled along all other male and female siblings who were within 8 years of the MTC. Low-risk families were also recruited from the same neighborhoods as the high-risk families, with the same family composition structure but without a drunk driving conviction or AUD diagnosis for either parent. A third subset of moderate-risk families was also enrolled; this involved families from the same neighborhoods, but where a parent may have met the AUD diagnostic criteria but never had a drunk driving conviction. As a result of these recruitment strategies, the adolescent sample is predominantly male (70.5%) and White (89.9%). For a full description of the sample and study procedures (see [53]).

### 2.2. Procedure

MLS assessments were completed in 3-year increments starting with 3–5-year-olds at Wave 1 (e.g., Wave 2, ages 6–8). Using these data, the association of ACEs on SU in adolescence through internalizing symptoms was examined. ACEs were measured during Waves 1–3 (ages 3–11), internalizing symptoms during early adolescence (Wave 4, ages 12–14), and SU during later adolescence (Wave 5, ages 15–17). The timing of these different assessments is largely in line with developmental research and pediatric recommendations indicating that ~2 years to 11 years represents childhood [54,55], that internalizing symptoms tend to emerge and increase through adolescence [56], and that 15 years is generally when the onset of SU tends to happen [57] on average. Parent and self-reports were used for ACEs and internalizing symptoms. While self-report bias may have occurred, given that the adolescents were reporting about themselves, it is important (and aligned with trauma-informed principles) to understand the lived experiences of the participants [58]. Informed consent and assent were obtained from parents and children after study procedures were reviewed. This study was approved by the internal review board where the study took place.

### 2.3. Measures

#### 2.3.1. ACEs

A cumulative measure of ACEs during childhood (prior to age 11) was used, consistent with prior work [51], and was constructed by grouping items across several questionnaires. Five items were taken from the Oregon Social Learning Center Family Crisis List [59]. In this measure, caregivers mark crises the family experienced in the past 6 months. Items used for the current study reflected the following: not being able to pay bills, lacking clean clothes, a family member seeing a mental health professional, something stolen from the house, and applying for welfare or unemployment. Eight items were taken from the Conflict Tactics Scale (CTS; [60]). The CTS assesses family violence by asking caregivers about methods of conflict resolution among family members in the past year. Items used for the current study reflected caregiver behaviors toward their child, such as insults, threatening to or hitting or throwing something at their child, and hitting their child. To assess the child’s exposure to domestic violence, items reflecting these same behaviors, but directed toward the parent and their spouse, were used. Additionally, to assess sexual or physical abuse prior to age 11, an adolescent retrospective self-report measure of the CTS was used. Items were also taken from a modified version of the Coddington Family Events Questionnaire [61]. This parent-report measure assesses life events that occurred in the family during the past 3 years. Items used for the current study reflected the following: the death of a sibling, parent incarceration, sibling involvement with SU, and bullying by classmates. Finally, one item from the fourth version of the Diagnostic Interview Schedule [62] was included that reflected parental AUD. In total, 21 items were aggregated to derive a measure of ACEs (α = 0.61). All of the items in the ACE score were first dichotomized to reflect whether the event occurred (1 = yes and 0 = no). If an item was endorsed at multiple time points, the event was still coded as 1 (i.e., max value = 21) rather than “double counting” an event, consistent with prior work (e.g., [17,48]).

#### 2.3.2. Internalizing Symptoms

Separate measures of depression, anxiety, and somatic symptoms at Wave 4 (ages 12–14) were created based off the work of [63] using the Youth Self Report (YSR; [64]) and the Child Behavior Checklist (CBCL; [65]). This alternate method of scoring is more closely consistent with current diagnostic symptomology for anxiety and depression. Reports of these three symptoms from the YSR and CBCL were tested in separate models to account for potentially differing effects between self- and parent-reported data. Items on the YSR and the CBCL are scored on a scale from 0 to 2 (0 = not true and 2 = often true). Measures of depression, anxiety, and somatic symptoms consisted of 7, 12, and 6 items, respectively, for both parent and adolescent self-reports. Internal consistency was adequate and the variables were normally distributed (parent-reported internalizing symptoms [range of αs = 0.63–0.73; range of skewness = 1.6–1.9; range of kurtosis 2.7–4.9] and self-reported internalizing symptoms [range of αs = 0.70–0.79; range of skewness = 1.1–1.6; range of kurtosis = 1.2–3.3]).

#### 2.3.3. Substance Use (SU)

SU at Wave 5 (ages 15–17) was assessed via adolescent self-report using the Drinking and Other Drug Use Form [66]. To assess problematic alcohol use, an item assessing the maximum number of alcoholic beverages consumed in a 24-h period in the past year was used. To assess cigarette and marijuana use, an item assessing the frequency and occasions of use during the past year was used. Variables were normally distributed (range of skewness = 1.6–1.7 and kurtosis = 1.3).

#### 2.3.4. Control Variables

All of the models controlled for race (0 = non-White and 1 = White), biological sex (0 = male and 1 = female), and age at Wave 5. Additionally, the models were assessed both with and without externalizing symptoms at Wave 1 as a control variable to determine whether the effects held above and beyond the externalizing pathways. Externalizing symptoms were assessed using [63] a subscale for oppositional-defiant symptoms from the CBCL (α = 0.73, skewness = 0.7, and kurtosis = 0.2).

### 2.4. Data Analysis

Path analysis models were estimated in Mplus 8.1 [67] using full-information maximum likelihood to maximize the available data. In total, the models examined two reporters (parents and adolescents), three substances (alcohol, cigarettes, and marijuana), and with and without externalizing symptoms as a covariate, resulting in 12 total models. To account for the inclusion of siblings in the sample, multilevel analyses accounting for family clustering and controlling for biological sex, race, and age were estimated.

Mplus offers two options to evaluate mediated effects. The first approach is the product-of-coefficients method using the IND command. The second option calculates bias-corrected bootstrap confidence intervals (BBCIs), which is considered to be more robust [64]. In Mplus 8.1, clustering cannot be accounted for while using resampling methods. As a result, indirect effects when controlling for family cluster effects were compared to BBCIs, which has been performed in prior work (e.g., [68]). The results were mainly the same across both methods. However, we present path estimates calculated using 95% BBCIs (with 10,000 bootstrap samples), as these indirect effects are considered more robust. The following fit statistics were examined for the model fit: the root mean square error of approximation (RMSEA), the standardized root mean square residual (SRMR), the comparative fit index (CFI), and the Tucker–Lewis index (TLI). Low RMSEA and SRMR values (<0.08) represented a good model fit, whereas high CFI and TLI values (>0.80) represented a good model fit.

## 3. Results

Means, standard deviations, and correlations for all study variables are presented in Table 1. Adolescents in this sample experienced an average of four ACEs (range 0–12), with parents being involved in domestic violence (89%), parents pushing or grabbing their child (69%), and parents insulting their child (61%) being the top three ACEs experienced. Male and white adolescents experienced higher levels of ACEs. Females reported higher levels of all internalizing symptoms, and white adolescents reported higher levels of problematic alcohol and cigarette use. As expected, ACEs were associated with higher levels of externalizing symptoms, internalizing symptoms, and adolescent SU. Parent-reported depressive symptoms were associated with higher levels of cigarette use. Finally, self-reported internalizing symptoms were associated with higher problematic use of all substances.

### 3.1. Alcohol

**Figure 1:**
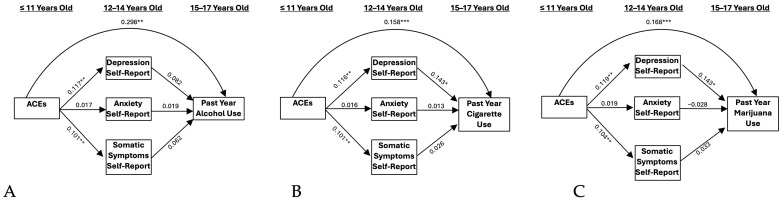
Self-report models. (**A**) Past Year Alcohol Use, (**B**) Past Year Cigarette Use, and (**C**) Past Year Marijuana Use. Note: ACEs = adverse childhood experiences. * *p* < 0.05, ** *p* < 0.01, *** *p* < 0.001.

**Figure 2:**
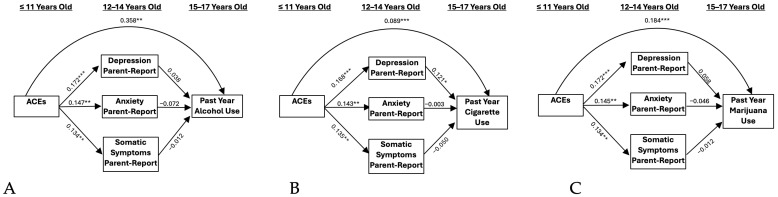
Parent-report models. (**A**) Past Year Alcohol Use, (**B**) Past Year Cigarette Use, and (**C**) Past Year Marijuana Use. Note: ACEs = adverse childhood experiences. * *p* < 0.05, ** *p* < 0.01, *** *p* < 0.001.

**Figure 3:**
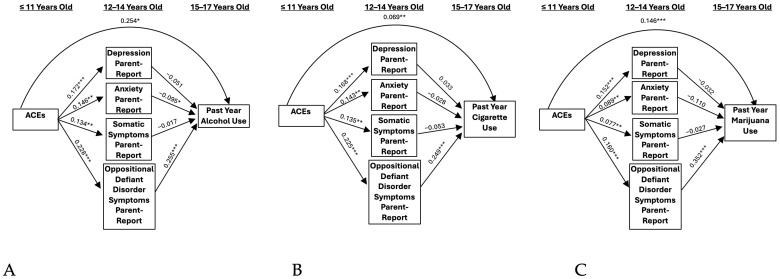
Parent-report models including externalizing symptoms. (**A**) Past Year Alcohol Use, (**B**) Past Year Cigarette Use, and (**C**) Past Year Marijuana Use. Note: ACEs = adverse childhood experiences. * *p* < 0.05, ** *p* < 0.01, *** *p* < 0.001.

Self- and parent-reported internalizing symptomology did not significantly mediate the relationship between ACEs and problematic alcohol use (Figure 1A and Figure 2A). However, when including externalizing symptomology as a covariate, a significant indirect effect resulted through parent-reported anxiety symptoms (IE = −0.022 and BBCI [−0.068, −0.001], Figure 3A). This association was negative, indicating that ACEs were associated with greater anxiety, which, in turn, predicted lower alcohol use. A one standard deviation unit increase in ACEs resulted in a decrease of −0.014 standard deviation units in alcohol use through its effect on parent-reported anxiety.

### 3.2. Cigarettes

**Figure 4:**
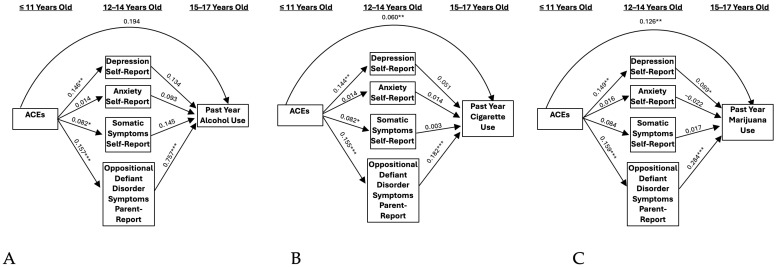
Self-report models including externalizing symptoms. (**A**) Past Year Alcohol Use, (**B**) Past Year Cigarette Use, and (**C**) Past Year Marijuana Use. Note: ACEs = adverse childhood experiences. * *p* < 0.05, ** *p* < 0.01, *** *p* < 0.001.

Self- and parent-reported depressive symptoms significantly mediated the relationship between ACEs and cigarette use (IE = 0.009 and BBCI [0.002, 0.022]; IE = 0.011 and BBCI [0.002–0.027]; Figure 1B and Figure 2B, respectively). These results indicate that ACEs predicted higher levels of depressive symptoms, which, in turn, predicted higher levels of cigarette use. A one standard deviation unit increase in ACEs resulted in an increase of 0.017 standard deviation units in cigarette use through its effect on self-reported depression. Additionally, a one standard deviation unit increase in ACEs resulted in an increase of 0.020 standard deviation units in cigarette use through its effect on parent-reported depression. When adding externalizing symptoms as a covariate, significant indirect effects remained only for the self-report model (IE = 0.007 and BBCI [0.001, 0.019], Figure 4B), but not the parent-report model. Accordingly, a one standard deviation unit increase in ACEs resulted in an increase of 0.014 standard deviation units in cigarette use through its effect on self-reported depression.

### 3.3. Marijuana

Self-reported depressive symptoms significantly mediated the relationship between ACEs and marijuana use (IE = 0.016 and BBCI [0.003–0.040], Figure 1C). This model indicates that ACEs predict higher levels of depressive symptoms, which, in turn, predicted higher levels of marijuana use. A one standard deviation unit increase in ACEs resulted in an increase of 0.017 standard deviation units in marijuana use through its effect on self-reported depression. Results were not significant for the parent-report model (Figure 2C). When adding externalizing symptoms as a covariate, significant indirect effects remained for the self-report depression model (IE = 0.013 and BBCI [0.001–0.036], Figure 4C). A one standard deviation unit increase in ACEs resulted in an increase of 0.014 standard deviation units in marijuana use through its effect on self-reported depression.

## 4. Discussion

In the current study, we examined the role of internalizing symptoms as a mediating pathway between ACEs and problematic SU in adolescence. To do so, we employed a novel approach to data analysis. Specifically, we (1) examined depressive, anxious, and somatic symptoms separately in the same statistical model (separate models for different substances) to tease apart the relative impact of these different symptom sets, (2) included adolescent and parent reports of internalizing symptoms in the same model, and (3) examined all models (i.e., alcohol, cigarette, and marijuana use) with and without externalizing symptoms as a control variable. As hypothesized, depressive symptoms mediated the association between ACEs and adolescent SU; however, anxious symptoms were only a significant mediator for the association between parent reports of anxious symptoms and alcohol use, and somatic symptoms were not significant mediators in any of the statistical models. Additionally, findings were generally consistent when externalizing symptoms were included and excluded in the statistical models—a critical finding, since externalizing symptoms are typically viewed as the primary etiological pathway to adolescent SU [69,70,71].

### 4.1. Study Findings

Decades of research support the co-occurrence of SU and mental health problems (see the recent review by [72]), as well as a graded association between ACEs and more severe problems (e.g., [4]). Additionally, the ACEs framework highlights the ubiquity of childhood adversity and its contribution to maladaptive outcomes, including SU and mental health problems [4,9,73]. Thus, SU and mental health concerns are likely to go hand-in-hand, especially for youth who have a history of ACEs. It is therefore important to consider all of these constructs within the same statistical model to inform prevention, assessment, and intervention efforts capable of addressing these issues concurrently.

Our findings related to the nuances in internalizing processes are in line with the existing literature. That is, we did not find somatic symptoms to be a significant mediator between ACEs and adolescent SU; however, depressive and anxious symptoms were statistically significant mediators in the current study. This is similar to other studies which have found more empirical support for the association between SU and depressive or anxious symptomology [19] compared to somatic symptoms [74]. This may be because adolescents who experience somatic symptoms (e.g., headaches or body pain) are less likely to engage in SU due to concerns about the physical effects of SU (e.g., hangovers and dependence), which they may believe would exacerbate these somatic symptoms. Additionally, our findings indicate that depressive symptoms may represent a riskier pathway to adolescent SU compared to anxious symptoms. We found depressive symptoms to be a significant mediator for two (i.e., cigarettes and marijuana) out of three of the substances we considered; anxious symptoms were only significant for one substance (i.e., alcohol). Previous research efforts also spotlight associations between ACEs, depressive symptoms, and SU. For example, Elmore and Crouch [75] reported that all but one of the individual ACEs they assessed had stronger associations with depression than anxiety. Another group of researchers [76] assessed the effects of ACEs on four subtypes of symptom composition (anxiety only, depression only, comorbid, or none); they found stronger results for the depression-only and comorbid groups compared to the anxiety-only group. Indeed, past work supports a more consistent association between depressive symptoms and SU compared to anxious symptoms [17]; however, there have been limited efforts to examine the unique domains of internalizing problems as distinct constructs within the same statistical model. Taken together, our differential findings (i.e., depressive pathways vs. anxious pathway) and the ACEs framework may indicate that, for some adolescents, depression symptoms have a stronger or comorbid role to play in the development of SU resulting from ACEs when compared to anxious symptoms alone or somatic symptoms.

Another layer of findings within the current study emerged because of our multi-informant approach to data collection and analysis. Our results varied by reporter (i.e., the adolescent or parent providing the data), with adolescent self-reported symptoms contributing to four significant models and parent-reported symptoms only contributing to two significant models. More specifically, adolescent self-report of depressive symptoms was a significant mediator between ACEs and cigarette and marijuana use with and without the inclusion of externalizing problems in the statistical model. However, the parent report of depressive symptoms was only a significant mediator between ACEs and cigarette use without including externalizing problems in the model. One reason for this pattern of findings may be that parents can be aware of observable depressive symptoms (e.g., disruptive behavior and defiance) that overlap with externalizing behavior problems, but other depressive symptoms are more internal and private to the adolescent (e.g., mood, feelings, and thoughts; [77]). Therefore, if an adolescent is choosing to smoke cigarettes to deal with internal (i.e., invisible and therefore unnoticed by parents) depressive symptoms, it stands to reason that only adolescent-reported symptoms would significantly mediate the association between ACEs and cigarette use. In contrast, adolescent self-report of anxious symptoms was not significant in any of our models; however, the parent report of anxious symptoms was a significant mediator between ACEs and alcohol use, but only when we included externalizing problems in the statistical model. That is, greater parent-reported anxiety served as a protective factor between ACEs and problematic alcohol use when controlling for externalizing symptoms. Compared to internal depressive symptoms, parents may be better able to observe and report visible symptoms of anxiety in their children (e.g., anxious nail-biting; [78]). As to why this result only emerged when externalizing symptoms were included as a control, these results could indicate that only adolescents with observable and high levels of anxiety (i.e., when controlling for potentially comorbid externalizing symptoms) are risk-averse and do not view alcohol as a viable means to manage their symptomology. Therefore, this high level of “pure” anxiety (i.e., observable anxious symptoms without comorbid externalizing symptoms) as indicated through parent reports could have protective effects against alcohol use via the aversion to the negative consequences of alcohol use [79]. It could also be true that, since the adolescent’s anxiety is so high and visible, their parent is involved, aware, and attentive to their needs, thus mitigating the adolescent’s use of alcohol. This set of findings supports the use of multi-informant data and our hypothesis that adolescent self-reported symptoms would have a stronger effect on the association between ACEs, internalizing symptoms, and adolescent SU. It is important to note that our interpretation of these findings does not determine whether adolescent self-report or parent report is more accurate, but rather that both offer different vantage points that can be relevant depending on the variable of interest.

A third layer to our findings relates to the different types of SU we evaluated. In line with our hypotheses, ACEs appear to increase the risk for cigarette and marijuana use during late adolescence due to greater self-reported depression in early adolescence. Indeed, adolescents may use substances to self-medicate or cope with depression resulting from early stressors in childhood [37,80,81]. One group of researchers [82] posit that self-medication may be more relevant to those experiencing depressive symptoms compared to anxious symptoms due to the risk associated with SU during adolescence. That is, some adolescents experiencing anxiety symptoms may consider SU a precarious choice that would only serve to increase their negative emotions (i.e., anxiety) as opposed to alleviate them. Indeed, in a longitudinal study of 4536 young adults (18–25 years old), researchers observed a positive association between depressive symptoms and increased risk for the onset of cigarette and electronic nicotine delivery system (ENDS) dependence [83]. To understand their results, these researchers turned to a biological rationale. Namely, nicotine and other ingredients in cigarettes and ENDS help to modulate the monoamine system, which is the same system targeted by medications used to treat depression by enhancing dopamine in the reward system, and, by inhibiting monoamine oxidase, serotonin levels are increased. Serotonin plays a crucial role in mood regulation, happiness, and the overall sense of well-being. Despite age differences between samples, young adults in Thomas and colleagues’ [83] study were developing a new pattern of SU, and adolescents in our study were also likely to be new to SU given their younger age. Additionally, depressive symptoms can manifest as isolation, and cigarette smoking (more so than other substances) can be a solitary behavior [84]. Findings from previous research suggest that solitary substance use is more likely to be a maladaptive coping technique to deal with negative emotions [84,85]. In another example, a longitudinal pathway from family violence (i.e., a singular ACE) to adolescent SU (i.e., the general overall SU score, inclusive of alcohol and other drug use) was found to operate through depressive symptoms [80]. Thus, by examining distinct categories of internalizing symptoms within the same statistical model, our findings go beyond previous work and make a novel contribution to the field by demonstrating that ACEs contribute to the risk for adolescent cigarette and marijuana use primarily through subsequent depressive symptomatology compared to other internalizing clinical presentations.

In contrast to our cigarette and marijuana models, the results from our alcohol model revealed that greater parent-reported anxiety served as a protective factor between ACEs and problematic alcohol use when controlling for externalizing symptoms. Compared to smoking, which is more likely to occur in isolation, alcohol is considered the most normative and social type of SU among youth due to social drinking [84]. Higher levels of anxiety may be protective because anxious adolescents could be less likely to engage in social activities where drinking occurs due to fear of socializing or the fear of experiencing negative consequences [86]. Relatedly, we may not have tapped into the risky (vs. protective) effects of drinking because alcohol use is considered normative during adolescence. Indeed, adolescents are more likely to report intentions to use alcohol compared to cigarettes, with research indicating that teens associate drinking with the image of someone who “fits in” and smoking with someone who is rebellious, therefore hinting at the casual attitudes youth may have towards alcohol use [87]. Overall, our findings support the co-occurrence of SU and mental health problems [72] and the ACEs framework [4]. As others report [10], ACEs do seem to contribute to a greater risk of adolescent SU across different substances, and this risk appears to operate through an adolescent’s mental health. Thus, SU and mental health concerns are likely to go together, especially for youth who have a history of ACEs. These findings underscore the importance of a trauma-informed approach [88] to research, assessment, and prevention and intervention programming.

### 4.2. Clinical Implications

Adolescent SU, ACEs, and mental health problems in adolescence are all critical public health issues seriously affecting youth today. Even though it is well-known that ACEs increase the risk of poor mental health and SU problems, this knowledge has not yet been translated into evidence-based integrated treatments for youth [89]. Building off the results of this study, demonstrating how ACEs and mental health work in concert to affect SU, we join others in the call for trauma-informed integrated SU treatments [10,58,88,90] for adolescents.

A trauma-informed approach requires an understanding of the high prevalence of trauma, its ubiquitous impact on development, the importance of avoiding re-traumatization, the value of person-centered care, and the awareness and support of resilience [91,92,93]. The core principles of this approach are safety, trust and transparency, collaboration and mutuality, and empowerment, choice, and voice [88,94,95]. Others [96] also advocate for additional principles, such as screening for trauma, post-traumatic growth and healing, and access to knowledge, services, and resources as relevant to positive health-promoting approaches to trauma-informed care and practice. The authors of a recent systematic review and meta-analysis found empirical support for the use of trauma-informed interventions to reduce the symptoms of co-occurring post-traumatic stress disorder (PTSD) and SU; however, they noted that the majority of studies considered in the review included adult samples, while only one pilot study with adolescent participants was included in the review [97].

As evidenced by the findings of the current study, adolescent SU prevention and intervention programming efforts must consistently incorporate the joint impact of childhood adversity and internalizing symptomology, assessing for ACEs and traumatic experiences, mental health, and SU as routine practice [58,98]. This would help to contextualize the unique challenges facing youth and shift substance use treatment from a reactionary approach to a preventative one along the developmental trajectory. For example, participants in the MLS with a history of ACEs and depressive symptoms were at higher risk for cigarette and marijuana use than participants with other internalizing clinical presentations (i.e., anxious and somatic), highlighting important clinical markers for professionals to assess content areas for program developers to consider when creating prevention programs for adolescent SU. Additionally, it is important to note again that our findings differed depending on whether we analyzed adolescent self-report or parent report data. We observed (and hypothesized) that adolescent self-reported symptoms had a stronger effect on the association between ACEs, internalizing problems, and adolescent SU, even when we accounted for externalizing problems. While we do not make any claims about accuracy, these results do highlight that adolescent and parent reports offer different vantage points and underscore the importance of talking with youth about their lived experiences, health, and well-being. Routine ACEs screening is already being implemented in certain places (e.g., California) with a high acceptability from clients [99,100]—a practice that, on a larger scale, would be of great benefit. Currently, the treatments available to adolescents are underutilized by those with histories of ACEs and they have limited long-term efficacy [90]. Grounding integrated SU and mental health treatment in a trauma-informed approach opens the possibility for psychoeducation on both trauma and SU, life-skill-building, and peer support to be included in early prevention [101], as well as healthy coping and emotional regulation. These types of changes can support upstream prevention to address problematic SU among youth with histories of ACEs before they begin [10].

### 4.3. Limitations

Given the current dearth of literature on pathways to adolescent SU in the wake of ACEs, specifically when including internalizing symptoms as a mediator, these results make a significant contribution to the field. Nonetheless, there are limitations to note. First, although utilizing an ACE score is important to assess the cumulative effect of childhood adversity, it may be potentially inaccurate to utilize parent reports for a child’s ACEs due to factors like social desirability bias. Prior work has also shown that using retrospective data has limitations [10], but, unfortunately, attempting to use self-report data was not an option given that the MLS dataset does not provide adolescent self-reported ACEs prior to age 11. Future work on childhood adversity can combine official existing data, such as child report or Child Protective Services data, with parent report data to validate the reports of some ACEs and compile the most comprehensive representation of an adolescent’s adversity history.

Second, assessing the impact of cumulative ACEs is important to assess the joint effects of multiple adverse events; this approach has been supported by prior work [4,8,9]. The cumulative approach to measuring ACEs has received psychometric support [102]. However, there is also utility in understanding the individual effects of specific ACEs, as well as the commonalities between similar ACEs (e.g., the Dimensional Model of Adversity and Psychopathology [DMAP]; [103]). For example, it may be important to determine if one adverse event (e.g., child maltreatment vs. witnessing domestic abuse) is more likely to drive an effect or if ACEs comprising a similar dimension of adversity (i.e., threat vs. deprivation) differentially influence the trajectory of development and SU. Indeed, the DMAP approach has shown utility in understanding the developmental and neurological effects of ACEs, such that experiences of threat (e.g., sexual and physical abuse, medical trauma, and violence) tend to affect emotion reactivity and regulation, whereas experiences of deprivation (e.g., neglect, caregiver SU, and mental illness) tend to affect executive functioning [103]. Ultimately, the measurement approach taken to the experience of ACEs is likely goal-dependent, in that differences in approaches will serve different research aims. Additionally, the current study only assessed the occurrence of ACEs. Other researchers have reported that factors such as the timing (i.e., occurring in early childhood vs. late childhood; [104]) and chronicity (i.e., the duration of an adverse event; [105]) of ACEs may also be relevant. Future work may benefit from longitudinal designs that include these factors and assesses the proximal vs. long-term effects of ACEs.

Third, comorbid symptomologies were not assessed. Given that the focus of our work was to better understand how the individual effects of internalizing symptoms might act as mediators, anxiety, depression, and somatic symptomology were assessed separately. Internalizing symptomology, however, is highly comorbid, so it may be that two or all of the internalizing symptoms were experienced by adolescents in the sample in tandem. Furthermore, internalizing symptoms are also highly comorbid with externalizing symptoms [79], and while we did control for externalizing symptoms, future work should assess multiple facets of internalizing and externalizing symptoms and their interactive effects to reflect the comorbid symptomatology that may contribute to adolescent SU.

Our sample and the overall design may introduce some bias to our findings given that the youth were predominantly white and male. Given the research supporting differences across racial groups and biological sex with regard to ACEs, internalizing symptoms, and SU [20,106,107], it will be important that future work examine whether these findings can be replicated with more diverse samples. In addition, the timing of data collection for the original study (1985–2007) may impact our findings and dampen their representation of current SU trends. For example, traditional cigarette use is now less common with the rise of ENDS; however, we were not able to assess ENDS use in the current study. Study replication may clarify how trends impact the association between ACEs, internalizing symptoms, and adolescent SU.

## 5. Conclusions

The current study provides a more nuanced picture of internalizing symptoms, with the results indicating that depressive symptomology puts adolescents most at risk for problematic SU if they have experienced ACEs. Based on our findings that depressive symptomology was the strongest mediator in this association, early treatment of depressive symptoms should be considered of high clinical importance. This could significantly decrease the SU risk among adolescents with histories of ACEs. Therefore, we call for the development of trauma-informed prevention and intervention efforts for youth who have experienced ACEs and are struggling with mental health and SU problems. This requires that ACEs are (1) integrated into the models of SU prevention and intervention, and (2) recognized as powerful antecedents to later problems.

McLaughlin and colleagues [108] posit that transdiagnostic intervention (i.e., identifying psychological and neurocognitive processes related to trauma in childhood) is the best approach for those who have experienced ACEs. Key clinical approaches include strengthening social support systems and early screening and intervention by medical practitioners during primary care visits. Interventions targeting depressive symptoms and promoting monoamine normalization, such as physical activity, mindfulness, and psychotherapy [83], may also be effective in reducing or preventing nicotine dependence. Echoing the need for the recognition of ACEs in the context of adolescent SU, advocates of trauma-informed care and youth rights emphasize the importance of gathering input directly from the person who is experiencing symptoms [109]. Although the parent report is a reliable and often accurate method of collecting information, our differential findings based on the informant underscore the importance of offering adolescents a platform to freely discuss their behavioral health within trauma-informed organizations and trauma-specific clinical interventions.

## Figures and Tables

**Table 1:** Means, standard deviations, and correlations of the study variables.

	Mean	SD	1	2	3	4	5	6	7	8	9	10	11	12	13	14
Sex ^a^	0.29	0.45	-													
2. Race ^b^	0.89	0.30	−0.05	-												
3. Age W5	16.54	0.94	−0.02	**0.11**	-											
4. ACES W1–W3	4.21	2.43	**−0.18**	**0.09**	−0.01	-										
5. Externalizing symptoms W4	2.07	1.68	**−0.17**	0.01	−0.03	**0.26**	-									
6. PR depressive symptoms W4	1.61	2.16	−0.00	0.02	−0.01	**0.16**	**0.42**	-								
7. PR anxiety symptoms W4	1.04	1.49	−0.04	0.05	−0.03	**0.14**	**0.28**	**0.45**	-							
8. PR somatic symptoms W4	0.98	1.39	0.06	0.02	−0.00	**0.11**	**0.12**	**0.26**	**0.14**	-						
9. SR depressive symptoms W4	2.77	3.06	**0.11**	0.04	−0.03	**0.09**	**0.13**	**0.21**	0.07	−0.00	-					
10. SR anxiety symptoms W4	1.94	2.12	**0.11**	−0.00	−0.05	−0.00	0.06	**0.14**	**0.12**	0.00	**0.66**	-				
11. SR somatic symptoms W4	1.92	2.00	**0.19**	0.04	**−0.08**	0.05	**0.11**	**0.14**	**0.09**	**0.22**	**0.54**	**0.51**	-			
12. Alcohol use W5	4.44	6.73	−0.06	**0.12**	**0.26**	**0.12**	**0.22**	0.02	−0.03	0.00	**0.12**	**0.08**	**0.09**	-		
13. Cigarette use W5	0.72	1.31	0.03	**0.09**	**0.21**	**0.16**	**0.26**	**0.13**	0.06	0.01	**0.18**	**0.11**	**0.11**	**0.54**	-	
14. Marijuana use W5	1.23	2.27	−0.05	0.05	**0.22**	**0.19**	**0.26**	0.06	0.00	0.01	**0.14**	0.07	**0.08**	**0.65**	**0.61**	-

Note: ^a^ 0 = male, 1 = female; ^b^ 0 = other race, 1 = white; SR = self-report, PR = parent report, W = wave (ex. W3 = Wave 3, etc.); items in bold indicate significant correlations (*p* < 0.05).

## Data Availability

The original data presented in the study, Michigan Longitudinal Study, are openly available through the Institute for Social Research at the University of Michigan at https://www.icpsr.umich.edu/web/pages/ (accessed on 14 October 2024).
